# Low- and High-Tech Approaches to Control *Plasmodium* Parasite Transmission by *Anopheles* Mosquitoes

**DOI:** 10.1155/2011/891342

**Published:** 2011-08-17

**Authors:** Chris M. Cirimotich, April M. Clayton, George Dimopoulos

**Affiliations:** W. Harry Feinstone Department of Molecular Microbiology and Immunology, Johns Hopkins Bloomberg School of Public Health, 615 N. Wolfe Street, Baltimore, MD 21205, USA

## Abstract

Current efforts have proven inadequate to stop the transmission of *Plasmodium* parasites, and hence the spread of malaria, by *Anopheles* mosquitoes. Therefore, a novel arsenal of strategies for inhibiting *Plasmodium* infection of mosquitoes is urgently needed. In this paper, we summarize research on two approaches to malaria control, a low-tech strategy based on parasite inhibition by the mosquito's natural microflora, and a high-tech strategy using genetic modification of mosquitoes that renders them resistant to infection and discuss advantages and disadvantages for both approaches.

## 1. Introduction

Cyclopropagative development of *Plasmodium* parasites in their anopheline mosquito vectors is required for transmission between human hosts. During the first stages of this cycle, immediately following the ingestion of gametocytes by the female mosquito, extracellular parasites are exposed to a harsh environment in the mosquito midgut. Following progression to the ookinete stage in the midgut lumen, *Plasmodium* parasites invade the midgut epithelium at around 20 hours after ingestion and develop over approximately 10 days into a mature oocyst. Mitotic division leads to the production of thousands of sporozoites from a single oocyst, and these sporozoites are released into the hemolymph, at about 10–20 days after infected blood ingestion, depending on the *Plasmodium* species. At this stage, the parasites migrate to the salivary glands from where they can be transmitted to another host during a subsequent blood feed. Oocyst and sporozoite populations are severely compromised by mosquito-mounted immune responses, but the escape of a small proportion of parasites is sufficient for transmission to persist. With the increased resistance of *Plasmodium* to the current arsenal of drugs and *Anopheles *mosquitoes to insecticides and the lack of an efficacious malaria vaccine, it is clear that development of novel control strategies are crucial in order to reduce malaria transmission. Here, we discuss different methods to control transmission of malaria parasites via low-tech approaches using the mosquito's natural bacteria microflora or high-tech approaches involving the direct manipulation of mosquito genomes to render them resistant to *Plasmodium*.

## 2. Targeting *Plasmodium* Parasites through Mosquito Microbiota

Numerous surveys of mosquito midgut-associated bacteria (MAB) in laboratory and wild anopheline mosquitoes have been performed, and common bacterial genera (*Enterobacter*, *Pseudomonas*, *Pantoea*, and others) have been identified [1–8], with some of these bacteria closely associated with *Anopheles* mosquitoes [9–11].

A number of studies have shown that MAB impact the ability of *Plasmodium* parasites to develop to the oocyst stage in the mosquito gut tissue. Mosquitoes that have been treated with antibiotics to remove their MAB are more susceptible to *Plasmodium* infection, and reconstitution of the bacterial flora results in infections at the same level as untreated control mosquitoes [12]. When added to a parasite-laden blood meal, bacteria can interfere with parasite development [1, 2, 4, 5]. Interestingly, this interference appears to be exclusive to Gram-negative (G−) bacteria but is bacterial strain dependent, suggesting some bacteria possess an anti-*Plasmodium* property [1, 4]. However, no correlation between G bacteria presence and infection status was observed in field populations of *A. gambiae* and *A. funestus* from Kenya and Mali, although determination of the timing of bacterial and/or parasite acquisition by the mosquitoes was not performed [7].

Multiple mechanisms could result in the inhibition of parasite infection by the presence of bacteria. Lysis of trypanosomes [13] and *Leishmania* parasites [14] in triatomine bugs has been observed following biofilm formation on the parasite surface by *Serratia* bacteria (G−), while no such phenomenon has been described in *Plasmodium* infection of mosquitoes. Bacteria produce compounds with potential antimalarial properties (reviewed in [14]). It was recently identified that an *Enterobacter *bacterium isolated from wild mosquitoes in Zambia produces reactive oxygen intermediates that kill developing parasites in the midgut lumen, inhibiting *Plasmodium* prior to mosquito midgut infection [1]. Small populations of the bacterium can nearly eliminate ookinete formation in the midgut, providing proof of principle for the use of this and other bacteria to control malaria parasite transmission [1]. In general, G− bacteria show varying levels of inhibition at the early stages of parasite development, suggesting that diverse mechanisms of bacteria-mediated parasite inhibition exist [1]. 

Bacteria may play an indirect role in parasite interference through the induction of an anti-*Plasmodium* immune response in the midgut. Studies have suggested that the mosquito's anti-*Plasmodium* and antibacterial defense systems are largely overlapping. The mosquito gut microflora has been shown to stimulate basal immune activity, which in turn is acting against the malaria parasite [12, 15–17]. The immune deficiency (IMD) innate immune pathway, which is stimulated by the presence of G− bacteria and appears to be the primary immune pathway activated in the mosquito midgut, has been shown to control *P. falciparum* infection intensities through the expression of anti-*Plasmodium* effector molecules in multiple anopheline species [18, 19]. These molecules also control bacterial populations in the midgut, providing a direct link between antibacterial and anti-*Plasmodium* immunity [12, 15, 19]. 

Although the absolute mechanism of inhibition exhibited by most bacteria remains unclear, their potential use as a biological-based control strategy is apparent. Nonetheless, there are deficiencies in our understanding of the mosquito-bacteria interaction that needs to be resolved before such strategies can be implemented. A better understanding of the acquisition of MAB by wild mosquito populations is needed. Bacteria are necessary for larval mosquito development [20] but the mechanism of transmission of bacteria through immature stages of development to the adult mosquito midgut is controversial. Studies have shown that bacteria provided to larvae can be identified in adult midguts [5, 8, 21] but other studies suggest that transstadial transmission does not occur [22] and instead that MAB are derived from adult sugar and water sources [23]. However, adaptation of specific bacteria to effective colonization of the midgut is possible [24]. The interactions between mosquitoes and bacteria that lead to efficient colonization in the adult midgut must be understood for effective introduction of inhibitory MAB in wild mosquito populations in malaria endemic areas. 

Another important issue that must be resolved is the concentration of MAB required for efficient inhibition of parasite development in the mosquito. As little as 100 bacteria can significantly impact *P. falciparum* development when provided concurrently in a blood meal [1, 4] and MAB can inhibit oocyst formation when provided in sugar solution prior to parasite challenge [4, 19], but the concentration necessary for inhibition in natural settings is unknown.

## 3. Targeting Human *Plasmodium* Parasites Using Genetically Modified Mosquitoes

Germline transformation of *A. stephensi *was first reported in 2000 [25], and other important malaria vectors have since been transformed [26, 27]. In the process of transformation, a mobile genetic element is used to insert into the mosquito genome a gene of interest that is under the control of a specific promoter. Choice of promoters and effector genes are some of the most important factors for generating mosquitoes that are refractory to *Plasmodium* infection and in limiting the adverse fitness effects exerted by transgene expression. Genetic drive systems to integrate the transgene into wild mosquito populations are also essential for the implementation of genetically modified mosquitoes as tools for control of malaria transmission.

To target *Plasmodium *parasites during the developmental cycle, an effective anti-*Plasmodium* transgene must be expressed in a relevant tissue (midgut, fat body, and salivary glands) at a relevant time (when the parasite is present in that tissue). The promoter used for transgene expression will determine the timing and the mosquito tissue in which the transgene will be expressed. In anophelines, midgut-specific transgene expression has been achieved using the carboxypeptidase [28], peritrophin [29], *Antryp1*, and *G12* [30] promoters, the vitellogenin promoter has been used to drive transgene expression in the mosquito fat body [31], and the *apyrase* [32] and* anopheline antiplatelet protein *[33] promoters can drive transgene expression in the salivary glands. Conditional transgene expression in *A. stephensi* midguts under the control of the *SRPN10* promoter has also been shown [34]. 

 Expression of nonmosquito effector molecules in transgenic mosquitoes has been used to decrease *P. falciparum* development. Midgut-specific expression of a sea cucumber C-type lectin in *A. stephensi* [35] and a synthetic anti-*Plasmodium* peptide in *A. gambiae* [36] decreased oocyst intensities. Transgenic technologies have also been used to increase the expression of endogenous mosquito genes that in turn increase anti-*Plasmodium *responses in the mosquito. Overexpression of Akt, a key signaling molecule in the insulin signaling pathway, in the midguts of *A. stephensi* completely blocks *P. falciparum *oocyst development [37]. Because the *Anopheles* innate immune system is engaged at multiple stages of *Plasmodium* infection and mediated through multiple factors (reviewed in [38]), the transgenic overexpression of multiple anti-*Plasmodium* immune effectors in several tissues at different times during *Plasmodium *infection can provide tiers of inhibition, targeting parasites that may have escaped the first lines of defense and decreasing the likelihood of resistance developing in the parasite (Dimopoulos et al. unpublished). Stable and heritable RNA interference-mediated silencing of endogenous mosquito transcripts through the expression of a hairpin-loop transgene [39] might also be used to target negative regulators of anti-*Plasmodium* responses to increase resistance to parasite infection [18].

An effective genetic drive mechanism is needed to introduce anti-*Plasmodium *effector transgenes into a wild mosquito population. A drive mechanism should be powerful enough to spread the transgene to near fixation in the population, be tightly linked with the transgene so that separation cannot occur and have minimal impact on mosquito fitness. Potential drive mechanisms are naturally occurring “selfish” gene mechanisms with non-Mendelian inheritance (reviewed in [40]) including, but not limited to, transposable elements (TEs) homing endonuclease genes (HEGs) and *Medea*. 

TEs are mobile genetic elements that are capable of moving rapidly into populations and can be engineered to carry a transgene through a population. However, the rates of transposition for the class II transposons *Hermes*, *Minos*, *Mos1*, and *piggybac*, which have been vital for mosquito transgenesis, are not sufficient to serve as drive systems [41]. While TEs randomly integrate into a genome, HEGs use a specific DNA sequence to integrate into the chromosome through a mechanism of double-stranded DNA break repair. These enzymes are active in *A. gambiae *cells and embryos [42] and can also be engineered to carry specific DNA sequences. A breakthrough in mosquito-based genetic drive systems was recently achieved with the successful introduction of an HEG into transgenic anopheline mosquitoes [43]. In cage studies, it was shown that the genetic element could invade naïve mosquito populations rapidly and may provide a novel mechanism of genetic modification of wild mosquitoes [43]. *Medea*, or maternal-effect-dominant embryonic arrest, causes the death of all offspring that do not inherit the *Medea*-bearing gene [44]. In this system, there is maternal expression of a toxin regulated by a germline-specific promoter and only zygotes expressing an antidote to the toxin will survive. Studies in the fruit fly have shown that *Medea *can effectively and rapidly drive transgenes into a population [44]. As novel mosquito germline-specific promoters are discovered, such as DNA regulatory regions of the *vasa* gene [45], both HEGs and *Medea* will have tremendous potential as genetic drive systems in mosquitoes. 

In order for transgenic mosquito technologies to be successfully applied, the genetically modified mosquitoes must be able to compete with wild mosquitoes. Therefore, the transgenic mosquito must be reproductively fit to ensure that the transgene will fix in the population. When a fitness cost is observed, it is difficult to determine the origin [46]. The impact on mosquito fitness could be due to insertional mutagenesis caused by the integration of a transgene into an endogenous gene [47, 48], the expression of the transgene itself [29], or inbreeding repression due to rearing transgenic mosquitoes to homozygosity [47]. However, some studies show that transgenic mosquitoes are as fit as nontransgenic mosquitoes [49, 50]. Of note, a report by Marrelli et al. [51] suggested that transgenic mosquitoes expressing antimalarial effectors may have a fitness advantage over wild-type mosquitoes when under the selective pressure of continuous infection.

## 4. Comparison of Low- and High-Tech Control Strategies

Here, we compare some of the important attributes for an effective control mechanism that each of these strategies has.

**Mass Production**

In order to test for anti-*Plasmodium *activity in MAB, the microorganisms must first be grown in culture. Because of this, optimal bacterial candidates for eventual release could be produced in mass quantities with minimal supplies that could be housed in endemic countries. In contrast, mass production of mosquitoes requires large facilities for rearing and sex selection and large amounts of supplies.

**Storage and Transportation**

Bacteria can be freeze-dried [52] for both storage and transportation, making introduction into remote areas possible. This also suggests that MAB could be combined with current mosquito control strategies such as entomopathogenic fungi in an applicable formulation [53]. No protocols are currently available for the preservation of viable *Anopheles *eggs, making transportation of either larvae or adult mosquitoes necessary.

**Parasite Species Coverage and Selection Pressure**

Mosquitoes can develop resistance to the toxin of a current biocontrol bacterium (Bti) [54], but a bacterial product with *Plasmodium* species specificity would be required for the parasite to develop resistance. Population genetic studies suggest that refractoriness is a dominant trait and that *Plasmodium* infection is a result of immune failure [54–56]. This, combined with a general antibacterial immune response, suggests that the use of MAB to inhibit malaria parasite infection of mosquitoes would not impart a selective pressure on the parasite. Indeed, both *P. falciparum* and *P. vivax* infections can be inhibited by MAB [1, 2, 4, 5]. For transgenic mosquitoes, the transgene will determine the range of parasites that can be inhibited and the selective pressure imparted on the parasite. Current effector molecules may not impact human malaria parasites and transgenic activation of mosquito anti-*Plasmodium* responses has only been shown to inhibit *P. falciparum* [37]. The use of transgenic mosquitoes expressing an exogenous gene may be problematic in that the parasite could develop resistance, but this would be overcome using endogenous immune gene overexpression or multiple releases over time of mosquitoes carrying different transgenes.

**Mosquito Species Coverage**

The ability of a bacterium to colonize or survive in the mosquito midgut is dependent on the bacterium itself, whereas only mosquito species that have been transformed or those that are capable of hybridization with the transgenic species will be refractory to *Plasmodium* infection. However, the introduction of bacteria is dependent on the mosquito species found in the coverage area and the route of introduction, whereas transgenic mosquitoes can disperse and infiltrate the natural populations.

**Off-Target Effects**

Release of bacteria into an ecosystem could have effects on other organisms. Depending on the released bacterial species, there could be competition for resources with other essential bacteria within the environment or unintended mortality in nontarget insects and vertebrates. Assuming that the expressed transgene is not released from the mosquito body, one would not expect off-target effects from the release of transgenic mosquitoes.

**Time and Concentration Dependence**

Bacteria can be effective against *Plasmodium* in the midgut lumen and also during oocyst maturation, but this time window requires that bacteria be present prior to or soon after parasite ingestion. Also, variation in the concentration of inhibitory bacteria present in an individual mosquito can determine the efficiency of inhibition [1, 4]. Therefore, there is a strict correlation between the timing of bacterial introduction and parasite inhibition. With transgenic mosquitoes, the timing and level of transgene expression is controlled by a mosquito promoter and is experimentally determined prior to release of the mosquitoes into the field.

**Introduction into the Field**

Bacterial formulations are currently used for biocontrol of mosquito larvae. However, because of the unknown nature of the mosquito-bacteria interaction and the carriage of bacteria through immature stages to the adult stage (discussed above), the determination of application procedures for effective coverage of mosquito populations remains to be resolved. Also, the number of applications, bacterial concentration, and the size of coverage area are currently unknown. Transgenic *Aedes aegypti *mosquitoes have recently been released in field-based trials, but this technology utilized a female killing-based technique that reduces mosquito populations [57]. Mosquitoes with an expressed transgene have not been released as of yet, but could follow a similar strategy to that currently being employed. Transgene integration into a population has been shown experimentally in large-cage trials [58] but not in wild mosquito populations.

**Ethical Issues**

Release of genetically modified organisms has been under much scrutiny and a number of publications address these issues [59–61]. Among these issues are ethical concerns of education of potential risks in endemic countries, spread of the transgenic mosquito to other countries that have not agreed to the release, and coverage under the Cartagena Protocol [62]. Bacteria do not require genetic modification in order to exert an inhibitory effect on *Plasmodium *development in the mosquito and biocontrol formulations using bacteria are currently in use, so ethical issues of bacterial release may not be as great.

## 5. Conclusion

Even with tremendous research efforts and financial support to control transmission, malaria remains the most impactful vector-borne disease worldwide. As *Plasmodium* parasites and the mosquito vectors continue to develop resistance to effective drugs and insecticides, we must continue the development of novel strategies to interfere with the transmission cycle. We have briefly summarized two potential strategies based on one “low-tech” approach using the mosquito's natural midgut microflora and one more technologically-involved “high-tech” approach using transgenic mosquitoes refractory to parasite infection. Each has their advantages and disadvantages for eventual implementation, but in the future may be combined as part of an integrated control strategy for malaria transmission.
